# Porous silicon microcavities: synthesis, characterization, and application to photonic barcode devices

**DOI:** 10.1186/1556-276X-7-497

**Published:** 2012-09-03

**Authors:** Fernando Ramiro-Manzano, Roberto Fenollosa, Elisabet Xifré-Pérez, Moises Garín, Francisco Meseguer

**Affiliations:** 1 Centro de Tecnologías Físicas, Unidad Asociada ICMM/CSIC-UPV, Universidad Politécnica de Valencia, Av. Los Naranjos s/n, Valencia, 46022, Spain; 2 Instituto de Ciencia de Materiales de Madrid CSIC, Madrid, 28049, Spain

**Keywords:** Porous silicon colloids, Photoluminescence emission, Optical cavity modes

## Abstract

We have recently developed a new type of porous silicon we name as *porous silicon colloids*. They consist of almost perfect spherical silicon nanoparticles with a very smooth surface, able to scatter (and also trap) light very efficiently in a large-span frequency range. Porous silicon colloids have unique properties because of the following: (a) they behave as optical microcavities with a high refractive index, and (b) the intrinsic photoluminescence (PL) emission is coupled to the optical modes of the microcavity resulting in a unique luminescence spectrum profile. The PL spectrum constitutes an optical fingerprint identifying each particle, with application for biosensing.

In this paper, we review the synthesis of silicon colloids for developing porous nanoparticles. We also report on the optical properties with special emphasis in the PL emission of porous silicon microcavities. Finally, we present the photonic barcode concept.

## Background

Silicon is a key material in many industrial sectors as metallurgy, electronics, and photonics. Depending on the applications, different degrees of purity are used. It ranges from the metallurgical grade (MG), solar grade (SG), and electronic grade (EG) silicon. Most applications of MG silicon concerns bulk physico-chemical properties derived from its electronic structure (*sp*^3^-like bonding). Also, silicon is a semiconductor material, being nowadays the base material for electronics [1-3]. Finally, the huge refractive index (*n* = 3.5) value of silicon has allowed developing new optical devices as photonic crystals [4,5], waveguides, multiplexers [6], and nanolasers [7]. It is well known from the technology sector that silicon can grow spontaneously in the form of small particles as silicon powder [8]. Several groups have reported on the formation of silicon colloids. Korgel et al. [9,10] have developed sub-micrometric colloidal particles of amorphous silicon by the thermal decomposition of trisilane. Our research team has also developed silicon colloids [11] through chemical vapor deposition methods. They are spherical micro- and nanoparticles of polycrystalline [11], amorphous or porous silicon [12] with a diameter size between 0.5 and 5 μm. We have also shown that they work pretty well as optical microcavities in the visible [12] and infrared [11] regions of the optical spectrum. Porous silicon [13] also shows photoluminescence (PL) emission, and it can be used for sensing devices [14,15]. The PL emission of porous colloids resonates with the whispering gallery modes (WGM) of the microcavity resulting in a high PL intensity. The PL spectrum displays a unique photonic profile identifying each particle, which constitutes the basic idea of a new type of photonic bar encoding [16], similar to other previously reported encoding systems [17]. As porous silicon is a biocompatible material, such photonic barcodes are envisaged for applications in the fields of biology and medicine. In this paper, we will review the synthesis procedure of porous silicon colloids. Also, we will report on the optical properties (optical transmission and PL) of both particle ensembles as well as single particles. Finally, we will report on the concept of the photonic barcode concept based on porous silicon microcavities and its potential applications to biosensing.

## Methods

### Synthesis of silicon colloids based on chemical vapor deposition

**Figure 1:**
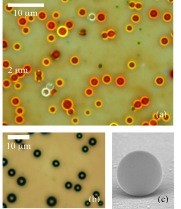
**Microscopy images.** (**a**) Optical image of porous silicon microspheres, and (**b**) optical image of a:Si-H microspheres. While porous silicon microspheres scatter yellow, orange, and red colors when irradiated by white light, a:Si-H microspheres look like black due to the absorption of visible light by silicon. (**c**) SEM image of a porous silicon microsphere of about 2 μm in diameter (reprinted with the permission of [12]).

The method for obtaining porous silicon microspheres is based on the decomposition of disilane gas (Si_2_H_6_) by chemical vapor deposition. The gas is introduced in a reactor whose walls are heated at high temperatures during certain time. During this procedure, Si_*n*_H_*m*_ clusters grow into the gas phase, and they become highly spherical micrometer-sized particles, thanks to surface tension forces. At the same time, there is a hydrogen desorption process from the clusters that makes the hydrogen content decrease progressively until they become hydrogenated amorphous silicon (a:Si-H) colloids. Depending on the decomposition time (DT), one can obtain amorphous [11] or porous silicon colloids [12]. Figure 1a shows an optical microscopy image at × 1,000 magnification of porous silicon microspheres obtained by a DT of 1 min and 30 s at 400°C. The particles are polydisperse in size with a diameter value from 0.5 to 5 μm, and they scatter red, orange, and yellow colors when irradiated by white light. For comparison, Figure 1b shows the optical microscopy image, at the same magnification as that of Figure 1a, of amorphous silicon microspheres that were obtained by the same decomposition temperature, but a bit longer DT, i.e., 5 min. In this case, no scattering of visible light occurs due to the absorption of silicon at these wavelengths, and particles look like black. Figure 1c shows a SEM image of a porous silicon microsphere that has a diameter of approximately 2 μm, illustrating its spherical perfection and its surface smoothness. An annealing treatment at 800°C for 1 h in vacuum was used to convert amorphous to polycrystalline silicon [11]. The high spherical perfection and the smooth surface of such particles allow them to scatter light very efficiently in a very broad spectral range covering the ultraviolet (UV), the visible (VIS), and the infrared (IR) range of frequencies [11].

## Results and discussion

### Optical properties of silicon colloids

**Figure 2:**
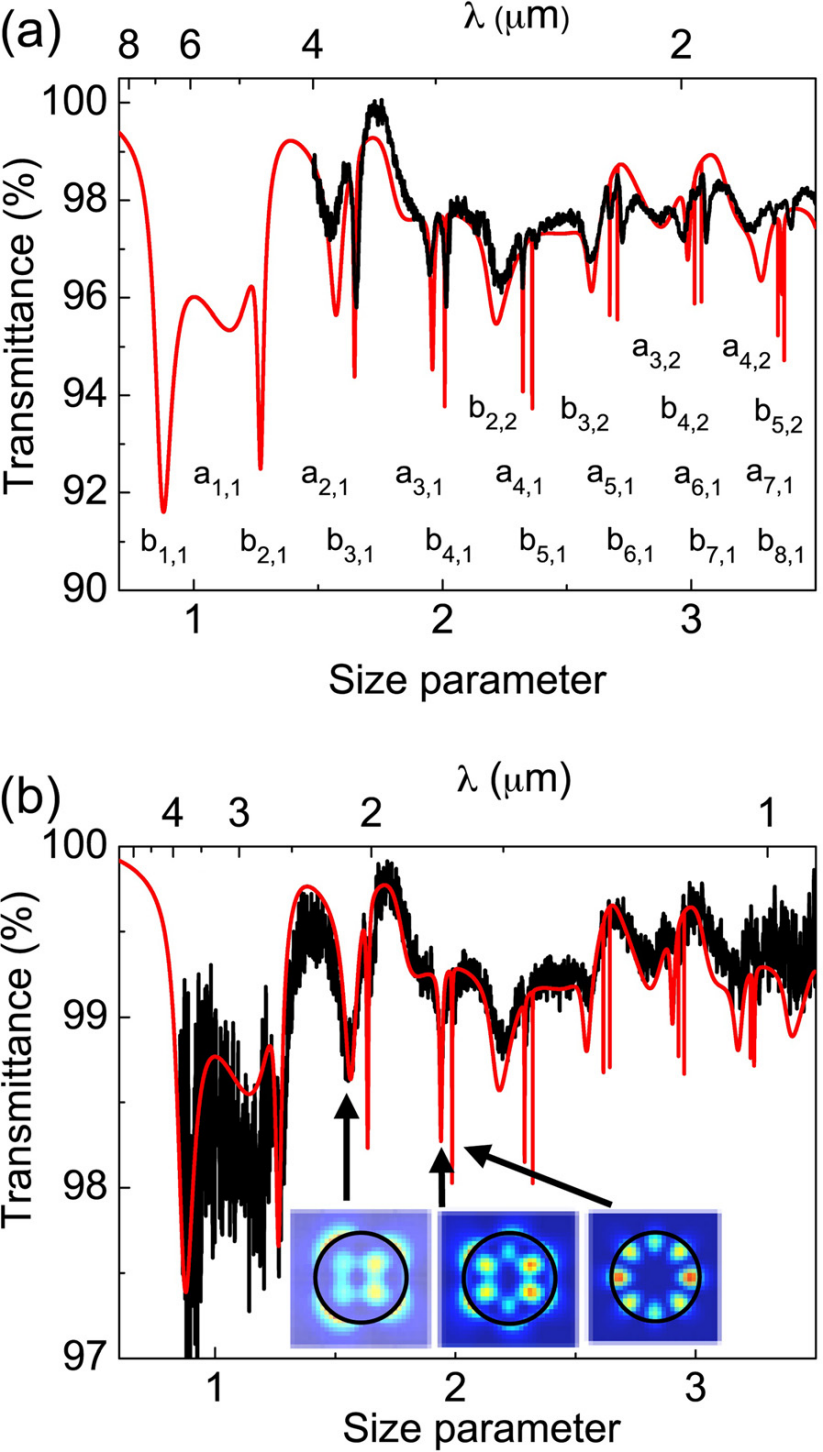
**Optical properties of polycrystalline silicon colloids.** Optical transmittance (black curves) and the Mie theory fit (red curves) of single silicon colloids of diameter *Φ* = 1,885 (**a**) and *Φ* = 1,050 nm (**b**). The dips of transmittance correspond to WGM. They are indicated under their corresponding dip in (**a**) by labels ‘b’ and ‘a’ for transversal electric and magnetic modes, respectively. The electric field intensity distribution is shown for modes (from left to right) a_21_, a_31_, and b_41_ in (**b**) (reprinted with the permission of [18]).

Figure 2 shows the optical transmission spectra (black curves) of two different polycrystalline silicon particles with a diameter of 1,885 (Figure 2a) and 1,050 nm (Figure 2b), as well as the theoretical fits (red curves) using the Mie theory. We used the refractive index values for crystalline silicon, with the particle diameter as the only fitting parameter [18]. Each dip in transmission corresponds to a resonating mode. They are indicated under their corresponding dip in the case of the larger microcavity (Figure 2a) by letters a (for transversal magnetic modes) and b (for transversal electric modes) and two sub-indexes that account for the different electric field intensity distributions. Figure 2b shows such distributions for modes a_21_, a_31_, and b_41_ (from left to right). Of special interest is the optical spectrum of the smaller microcavity. We have been able detecting the lowest modal number modes of the microcavity like b_11_ and a_11_, although they are still quite noisy. Low-index modes are very important because of two reasons: (a) the scattering cross-section is extremely large as they confine light very efficiently, and (b) their electric field distribution (see insets of Figure 2) is very similar to those of atomic orbitals. Silicon colloids also grow as particle ensembles forming a three-dimensional arrangement of spheres we call them as a photonic sponge [11]. These particle ensembles have very peculiar optical properties since they block very efficiently the UV, VIS, and IR radiations in a large wavelength region [11]. A 10-μm-thick coating of polydisperse silicon colloids is able to block 99% of the total sun radiation [11]. It may have important applications for both protecting from UV radiation as well as thermal proofing effects in cosmetics [19], paints, and coatings [20].

**Figure 3:**
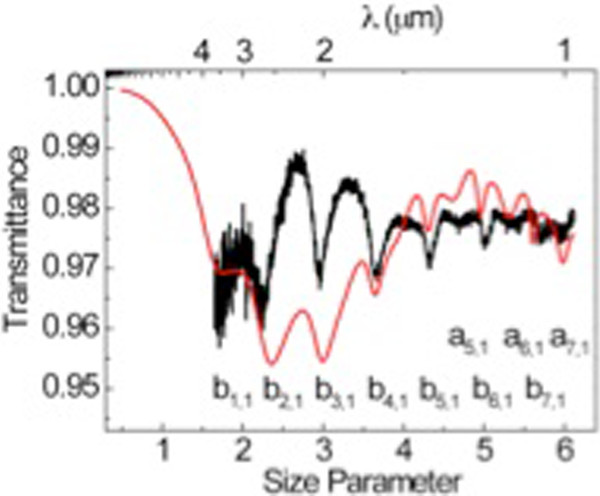
**Optical properties of porous silicon colloids.** Measured optical transmittance of a 1.910-μm-diameter porous silicon sphere (black curve). The Mie theory fit (red curve) gives a refractive index of 1.8 in all the measured range. The resonant modes are indicated under their corresponding deeps by letters ‘a’ (for transversal magnetic modes) and ‘b’ (for transversal electric modes) and two sub-indexes that account for the different electric field intensity distributions.

Figure 3 shows the optical transmission spectrum of a single porous silicon particle. The resonating modes of porous silicon appear in the near-infrared range. Different to the case of polycrystalline silicon colloids, the transmittance spectra of these particles could not be fitted to the Mie theory assuming a simple silicon colloid model with a homogenous value of the refractive index. The porous structure of silicon colloids may be not homogeneously distributed within the particle. In fact, the optical images of porous particles show an onion-like structure within the particle, suggesting a radial dependence of the porosity (see Figure 1). However, a reasonable fit could be achieved for small diameter spheres. This is the case of the spectrum displayed in Figure 3 (black line), which corresponds to a microsphere with a diameter value of 1.910 μm, and fabricated by a DT of 1 min and 10 s. The spectrum is plotted against the wavelength (*λ*) (upper axis) and size parameter (lower axis) that is defined as *π* × *Φ*/*λ*, *Φ*, and *λ* being the diameter of the particle and the wavelength value. The red line corresponds to the Mie theory fit. Such a fit was achieved by assuming a simple homogeneous distribution of the porosity within the particle and, therefore, a constant value of *n* = 1.8 in all the measured wavelength range. The agreement between the theory and experiment is reasonable as far as the mode position is concerned. However, some discrepancies between the theory and experiment regarding the absolute optical transmittance values are still present that could be ascribed to the porous silicon inhomogeneity of the microsphere.

**Figure 4:**
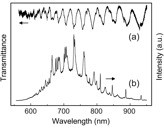
**Transmittance (a) and photoluminescence (b) spectra of a porous silicon microsphere of about 4.1-μm diameter.** Dips in the transmittance spectrum correspond to the peaks in the photoluminescence spectrum. They are associated to the optical resonances of the microcavity (reprinted with the permission of [16]).

The PL spectrum of a coating of porous silicon particles show the typical PL profile [12] shown in bulk porous silicon obtained through electrochemistry methods [14]. However, when we focus on the PL of a single particle, the spectrum changes completely. Figure 4b shows the PL signal of a 4.1-μm microcavity. Figure 4a shows the transmittance spectrum of the same microsphere. Dips in transmittance correspond to peaks in the PL spectrum. The intrinsic luminescence from porous silicon is strongly enhanced at wavelength values corresponding to the optical modes of the microcavity, resulting in a spectral response plenty of pronounced and narrow peaks, some of them with a full width at half maximum (FWHM) of the order of 1 nm. The PL features (Figure 4b) are much more pronounced and narrower than those obtained from optical transmittance (Figure 4a), this being a clear proof of microcavity-induced PL enhancement. Also, narrower peaks appear at the longer wavelength side of the PL spectrum where self-absorption is smaller. It should be mentioned that the optical resonance position blueshifts with the exposure of the microspheres to air due to an oxidation process as we have recently reported [12].

### Porous silicon colloids for biosensing: the photonic barcode concept

**Figure 5:**
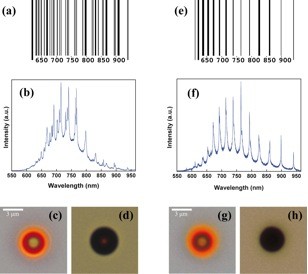
**Photonic barcodes (a and e) produced by two different porous silicon microspheres.** The location and width of the bars are determined by the position and FWHM of the resonances of their PL spectra (**b** and **f**, respectively). Optical microscopy images of the microspheres, taken at × 1,000 magnification in reflection (**c** and **g**) and in transmission (**d** and **h**) modes indicate, within the limited resolution of this technique, sphere diameter values of 4.1 and 3.7 μm, respectively (reprinted with the permission of [16]).

As porous silicon show PL emission in the transparent region of the biological tissue, they can be used as active particles for sensing applications [14]. The PL emission of a single porous silicon colloid (see Figure 5) is strongly coupled to the resonating modes of the microcavity. It results in a unique PL spectrum with a high quantum yield. This spectrum depends on two factors: the colloid size and the porosity value. The PL spectrum constitutes, therefore, a fingerprint of the particle. From these facts, we propose a photonic encoding procedure where a unique barcode can be assigned to each colloid by means of its PL spectrum. In the most common bar code, the Universal Product Code [21], the 1 and 0 binary numbers are represented by black bars and white voids whose width indicates the number of equal consecutive digits. Figure 5a,b and e,f illustrates how the optical spectra of two different porous silicon colloids, whose optical microscopy images in reflectance and in transmittance are shown in Figure 5c,d, and g,h, respectively, can be associated to two distinct photonic barcodes. One can see in Figures 5a,e that the different bars are located at the wavelength value of each resonance, and the bar width corresponds to the FWHM of its associated resonance. The bottom numbers of the barcode (see Figures 5a,e) indicate the wavelength scale.

**Figure 6:**
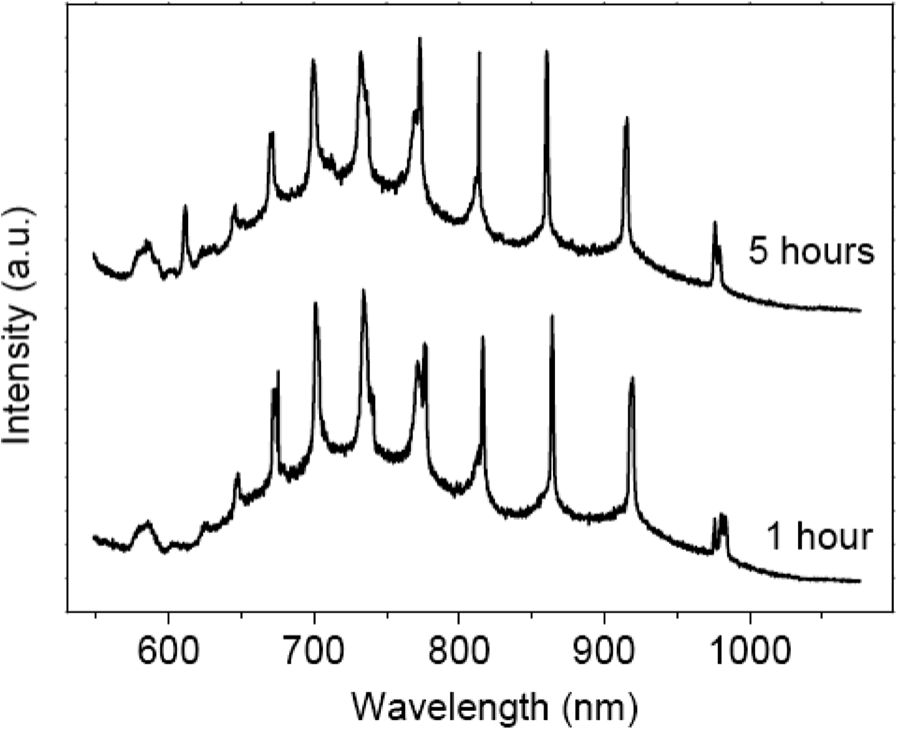
**PL spectra of an about 3.4-μm diameter porous silicon colloid.** After being immersed in 200-mM NADPH aqueous solution during 1 (bottom spectrum) and 5 h (top spectrum).

In the following, we will discuss on the potential application of porous silicon colloids as sensors for biomedicine and biology. In the last years, great interest has been paid to the development of micro- and nanometer-sized particles and systems able to act as photonic sensors [15,22,23] for medicine [24] and biology. As porous silicon-based sensors are biocompatible [15], stable [25], and biodegradable [26], photonic barcodes are envisaged for being used in the fields of biology and medicine. We have measured the PL stability of silicon colloids in biological agents such as NADPH aqueous solutions and bovine cultives. Figure 6 shows the PL spectra of a 3.4-μm-diameter porous silicon colloid after being immersed in a 200-μM NADPH-deoxidised aqueous solution for 1 (bottom spectrum) and 5 h (top spectrum). There are no significant differences between both spectra concerning both the overall PL intensity and the relative intensity of Mie PL peaks over the PL background (broad spectrum). Finally, the peak positions of the Mie-induced PL resonances remain unchanged over the period of time tested. Therefore, we can reasonably infer that porous silicon colloids are stable under the NADPH agent. The PL of porous silicon colloids is also stable under other biological environments like Roswell Park Memorial Institute medium with a fetal calf serum [16]. Finally, some of us, in collaboration with Canham et al., have also proved that amorphous silicon colloids quickly biodegrade in a few days [27]. The data suggests that amorphous silicon does not require nanoscale porosification for full *in vivo* biodegradability.

## Conclusions

We have reviewed the synthesis method we have developed for processing amorphous, polycrystalline and porous silicon colloids with diameter values between 0.5 and 5 μm. Because of both their spherical shape and micrometric size, all silicon colloid allotropes work pretty well as optical microcavities in the near-infrared region. Furthermore, the PL emission of porous silicon colloids is greatly enhanced when it resonates with the optical microcavity modes, resulting in a unique photonic fingerprint we call it as the photonic barcode.

## Competing interests

The authors declare that they have no competing interests.

## Authors' contributions

RF and FM designed the experiments presented in this work. RF processed the porous silicon colloids and preformed the scattering properties of particle ensemble. PL of single particles was performed by FRM and EXP. Theoretical calculations have been performed by EXP and MG. FM wrote the paper and all the authors revised it. All authors read and approved the final manuscript.
